# Monocyte Derived Microvesicles Deliver a Cell Death Message via Encapsulated Caspase-1

**DOI:** 10.1371/journal.pone.0007140

**Published:** 2009-09-25

**Authors:** Anasuya Sarkar, Srabani Mitra, Sonya Mehta, Raquel Raices, Mark D. Wewers

**Affiliations:** The Davis Heart and Lung Research Institute and the Division of Pulmonary, Allergy, Critical Care and Sleep Medicine, The Ohio State University, Columbus, Ohio, United States of America; New York University School of Medicine, United States of America

## Abstract

Apoptosis depends upon the activation of intracellular caspases which are classically induced by either an intrinsic (mitochondrial based) or extrinsic (cytokine) pathway. However, in the process of explaining how endotoxin activated monocytes are able to induce apoptosis of vascular smooth muscle cells when co-cultured, we uncovered a transcellular apoptosis inducing pathway that utilizes caspase-1 containing microvesicles. Endotoxin stimulated monocytes induce the cell death of VSMCs but this activity is found in 100,000 g pellets of cell free supernatants of these monocytes. This activity is not a direct effect of endotoxin, and is inhibited by the caspase-1 inhibitor YVADcmk but not by inhibitors of Fas-L, IL-1β and IL-18. Importantly, the apoptosis inducing activity co-purifies with 100 nm sized microvesicles as determined by TEM of the pellets. These microvesicles contain caspase-1 and caspase-1 encapsulation is required since disruption of microvesicular integrity destroys the apoptotic activity but not the caspase-1 enzymatic activity. Thus, monocytes are capable of delivering a cell death message which depends upon the release of microvesicles containing functional caspase-1. This transcellular apoptosis induction pathway describes a novel pathway for inflammation induced programmed cell death.

## Introduction

Caspase-1 was first described as the IL-1 converting enzyme responsible for processing and hence activating proIL-1β to its active form [1]–[3]. However, its structural homology to *C. elegans* death genes prompted the discovery of a class of proteases now termed caspases [4], [5]. It is now recognized that caspase-1 regulation depends upon the assembly of a protein complex, now termed the inflammasome. This structure is centered upon the adapter molecule ASC and typically another member of the NOD-like receptor or RIG-I receptor family that is thought to provide an intracellular danger sensing function [6]–[8]. Sensing of danger signals, either from pathogens (pathogen associated molecular patterns, PAMPs), from exogenous agents like silica, or endogenous signals such as ATP and uric acid, induces inflammasome assembly [6]. This assembly autoactivates caspase-1 by a proximity mediated process with the resultant release from the cell of processed IL-1β and IL-18 [9]–[12]. Interestingly, it is now generally recognized that with caspase-1 activation, not only is IL-1β and IL-18 processed and released by these danger sensing macrophages, but many of the inflammasome components themselves are also released, notably caspase-1 and ASC [13].

Although caspase-1 is generally categorized as an “inflammatory” caspase by virtue of its activation of IL-1β and IL-18, it is now clear that caspase-1 also plays a role in certain forms of cell death, e.g. sepsis induced lymphocyte apoptosis [14] and bacterial induced pyroptosis [15]–[17]. In these caspase-1 mediated processes, the dying cell's endogenous caspase-1 serves as the executioner caspase. However, in the process of analyzing how monocytes induce cell death in a co-culture model, we uncovered a novel transcellular mechanism of caspase-1 mediated cell death.

Monocytes sensing of danger signals induces the release of IL-1β, caspase-1 and ASC. These molecules are released in microvesicle packets that have the capacity to deliver a cell death message to proliferating smooth muscle cells. This cell death message is dependent upon the catalytic function of caspase-1 and upon the integrity of the microvesicle capsules. This novel pathway of caspase-1 mediated cell death adds a novel twist to the characterized functions of caspase-1.

## Results

### Contact independent apoptosis of vascular smooth muscle cells by monocytes

**Figure 1:**
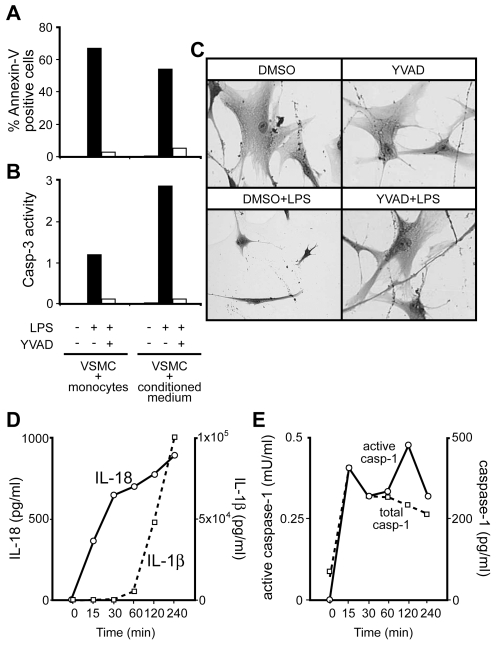
VSMC apoptosis by monocytes is contact independent and inhibited by caspase-1 inhibitor. Monocytes were stimulated with LPS (1 µg/ml) for 15 min in the presence or absence of YVADcmk. VSMCs were either co-cultured with these monocytes or subjected to conditioned medium from the stimulated monocytes. Apoptosis was measured by A) Annexin/PI and B) caspase-3 assays along with C) morphology. D) Monocytes were also stimulated with LPS (1 µg/ml) for indicated time and IL-18 and IL-1β release was measured from supernatants by ELISA. E) Supernatants were also analyzed for total and functional caspase-1 using ELISA and WEHD-assays. Cytokine and enzymatic assays depicted in graphs are based on average of n = 2 experiments.

Since monocytes take up residence with vascular smooth muscle cells (VSMCs) in atherosclerotic plaques [18]–[21], we used an *in vitro* co-culture model to analyze the monocyte ability to induce VSMC death. Vascular smooth muscle cells (VSMC) were cocultured with monocytes in the presence or absence of LPS (1 µg/ml) for 24 h. Monocytes stimulated with LPS induced significant VSMC cell death, as compared to VSMC cocultured with control unstimulated monocytes (**Figure 1A**). To determine whether this cell death induction of VSMC by monocytes was contact dependent, VSMC were cultured with conditioned medium from monocytes untreated or pretreated with LPS for 2 h. Cell survival was measured using crystal violet staining, total cell count under light microscope, Annexin V and caspase-3 activation assays. Conditioned medium from LPS treated monocytes resulted in significant VSMC cell death, whereas conditioned medium from control monocytes had no effect (**Table 1** **, Top panel**). Cell death was confirmed to be apoptosis as measured by classical apoptotic morphology of cells under light microscope (**Figure 1C**), Annexin V and caspase-3 activation assays (**Figure 1A and B** ). Apoptosis induction by monocytes was completely inhibited in the presence of the specific caspase-1 inhibitor, YVADcmk. Briefly, monocytes were pretreated with YVADcmk for 15 min, washed and then stimulated with LPS or left untreated for 2 h. Conditioned medium was then added to VSMC and effect on apoptosis was analyzed. YVADcmk completely abrogated apoptosis induced by LPS stimulated monocytes (**Figure 1**).

**Table 1:** Induction of VSMC death.

Experiment	Samples	Absorbance (570 nm)	P values
LPS stimulated	Control	0.89±0.13	
monocyte supernatant	LPS	0.45±0.08	0.0004*
Monocyte microvesicles	Control	0.83±0.13	
(pretreatment)	LPS + DMSO	0.38±0.05	0.003*
	LPS + YVAD	0.85±0.03	0.630*, 0.0001^#^
VSMC (pretreatment)	Control	0.32±0.03	
	YVAD	0.58±0.03	0.018*
	DEVD	0.78±0.05	0.010*
	ZVAD	0.89±0.02	0.003*
Monocyte microvesicles	Control	0.87±0.09	
(posttreatment)	Intact	0.43±0.05	0.004*
	Homogenized	0.85±0.04	0.86*, 0.013@
	Heat Inactivated	0.79±0.01	0.186*, 0.007^$^

ˆCell death of VSMC was measured at 18 h after treatments as outlined. Monocyte supernatants or MVs were all generated by 15 min. incubations with LPS (1 µg/ml). Vascular smooth muscle cell death was inversely related to the crystal violet staining of resident cells as measured at A_570_. **Top Panel**, VSMC death by conditioned medium from LPS stimulated monocytes (*LPS vs control); **Second Panel**, Inhibition of VSMC death by MV isolated from LPS treated monocytes that had been pretreated with the caspase-1 inhibitor, YVAD (*Treatments vs control, **^#^**YVAD vs LPS); **Third Panel**, VSMCs were pretreated with different inhibitors for 30 mins before being subjected to MV (*Inhibitors vs control); **Bottom Panel**, Effect of homogenization or heat-inactivation of MV before addition to VSMC (* Different MVs vs control, **^@^**homogenized vs intact, **^$^**heat inactivated vs intact).

### Cytokines and active caspase-1 release by LPS-stimulated monocytes

Conditioned medium from monocytes was analyzed for the presence of IL-1β and IL-18 by ELISA. Upon stimulation with LPS, monocytes released both mature IL-1β and IL-18 into the supernatant. IL-18 was measured in the medium as early as 15 min after LPS challenge. IL-1 β became detectable at 1 h and reached a peak at 2–3 h (**Figure 1D**). Release of both mature IL-18 and IL-1 β was completely abrogated in the presence of the caspase-1 inhibitor, YVADcmk. Caspase-1 was also identified in the supernatants of stimulated monocytes. Active caspase-1 was released as early as 15 min from LPS stimulated monocytes and this release increased in a time dependent manner (**Figure 1E**). That the released caspase-1 from LPS stimulated cells was active was confirmed by both immunoblot and WEHDafc enzymatic assay (**Figure 1E**).

### Microvesicular release of active caspase-1 and ASC by LPS-stimulated monocytes

**Figure 2:**
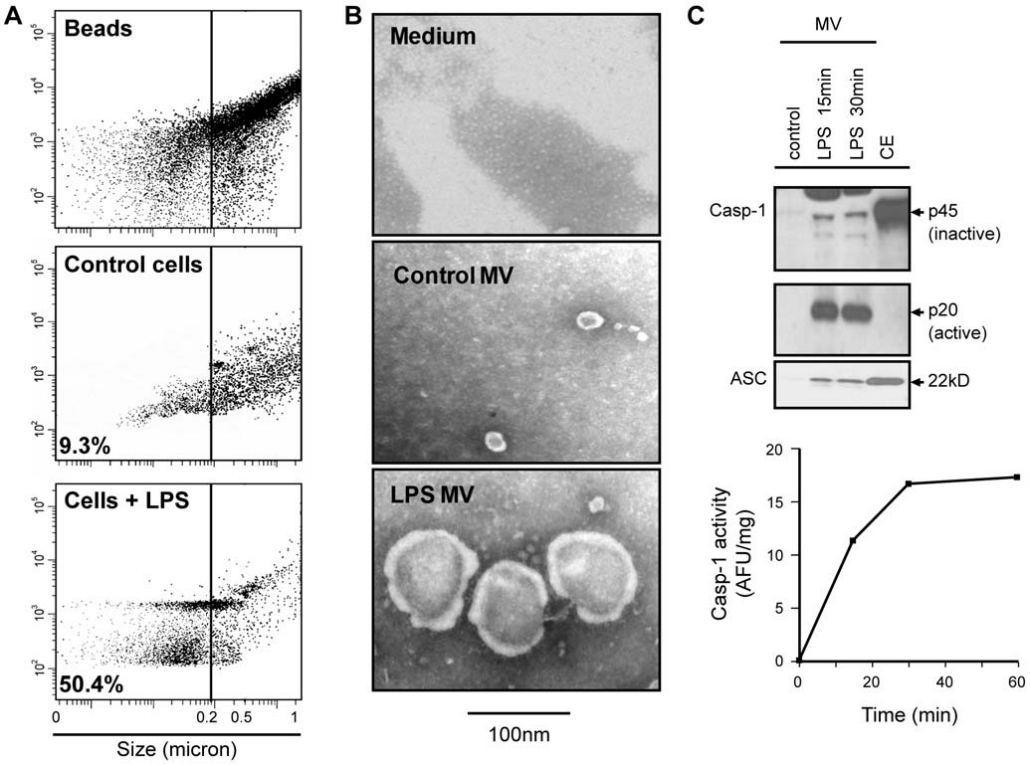
Active caspase-1 release from stimulated monocytes occurs in microvesicles. Monocytes were stimulated with LPS (1 µg/ml) for 15 min. Microvesicles were isolated from LPS stimulated monocytes supernatant by ultracentrifugation. Isolated microvesicles were analyzed by A) flow cytometry and B) transmission electron microscopy. C) Microvesicles were further analyzed for presence of catalytically active caspase-1 and inflammasome protein, ASC, by immunoblot. Activity of caspase-1 was also measured using WEHD-enzymatic assay. Immunoblots are representative of n = 3 experiments and caspase-1 enzymatic assay is average of n = 2 experiments.

We next examined the mode of release of active caspase-1 from stimulated monocytes freshly isolated from buffy coats. Microvesicle shedding is a recognized mode of cytokine release, as described for IL-1β and Fas ligand [22]–[29]. To examine the possibility of microparticle/microvesicle release microvesicles were isolated from unstimulated and LPS challenged fresh monocytes by ultracentrifugation. Isolated microvesicles were then quantified by flow cytometry. Stimulated monocyte microvesicles were enriched for smaller vesicles (i.e. <0.2 µm), 50.4% versus 9.3% for unstimulated monocyte microvesicles (**Figure 2A** **and** **Table 2**). Isolated microvesicles were also characterized by transmission electron microscopy. The LPS induced microvesicles were less than 0.1 micron (**Figure 2B**) by comparison to calibrated beads. That this microvesicle release by monocytes upon stimulation by LPS was not the effect of monocyte cell death was confirmed by analyzing the stimulated monocytes for cell death using Annexin/PI assay and LDH measurements from the supernatants (**Table 3**). These microvesicles were then subjected to immunoblot analysis for the presence of caspase-1 and ASC. Active microvesicular caspase-1 was shed from monocytes stimulated with LPS in a time dependant manner, as early as 15 min after LPS stimulation (**Figure 2C** **)**. This release of active caspase-1 in vesicles correlated with our earlier observation of active caspase-1 in the supernatants of monocytes stimulated for 15 min. That caspase-1 released in the vesicle was active was also confirmed by WEHDafc assay of the vesicles (**Figure 2C**). Caspase-1 release in MVs was also quantified, over time after LPS challenge, and compared to the amounts of caspase-1 in the cells. Approximately 15% of caspase-1 was released by the monocytes packaged in microvesicles as early as 15 min after LPS stimulation (**Table 4**). Monocytes pretreated with caspase-1 inhibitor, YVADcmk also released caspase-1 in vesicles upon stimulation, but in an inactive-proform. Microvesicles also contained the inflammasome protein ASC, critical for caspase-1 activation. ASC, similar to caspase-1, was identified both in the supernatants and microvesicles from LPS stimulated monocytes (**Figure 2C**).

**Table 2:** Monocyte microvesicle quantitation by flow cytometry.

*Samples*	*% Total*
	<0.2	>0.2–<1.0
Unstimulated MV	9	20
	12	17
	8	19
Stimulated MV	50	12
	58	12
	48	18

ˆEvents were quantified for 10,000 flow events of vesicles both <0.2 and >0.2–<1.0 microns from monocytes either untreated or stimulated with LPS (1 µg/ml) for 15 minutes. Data from n = 3 experiments.

**Table 3:** Microvesicle release by stimulated monocytes is independent of cell death.

Time (h)	Annexin-V assay (% positive cells)	LDH assay (% cytotoxicity)
0	0	0
0.25	4	3±2
0.5	4	3±1

ˆMonocytes were analyzed for cell death after stimulation with LPS for different periods of time. Annexin V assay (n = 2 experiments) and LDH (n = 3 experiments).

**Table 4:** Quantification of caspase-1 release in microvesicles.

*Time*	*Cell lysate (CE)*	*Microvesicles (MV)*
(h)	(ng/10^7^cells)	(ng/10^7^cells)
0	30	1±0.1
0.25	33±1	5±3
0.5	40±12	4±0.7
1	42±13	4±0.4
2	43±18	4±2

ˆMonocytes (10^7^/ml) were stimulated with LPS for indicated time and MVs were isolated from the supernatant. Cell lysates (CE) and MVs isolated from the supernatant were quantified for caspase-1 using ELISA.

### Monocyte microvesicular caspase-1 induces VSMC apoptosis

Vascular smooth muscle cells were then co-cultured with microvesicles isolated from conditioned medium of either unstimulated monocytes or monocytes stimulated with LPS for different periods of time. Monocytes used in all experiments were freshly isolated from buffy coats. Cell death of VSMCs was analyzed using Annexin V and crystal violet assays. Microvesicles isolated from as early as 15 min of LPS stimulation to monocytes induced approximately 43% cell death of VSMC as compared to control vesicles (**Table 5** **, Panel 1 and 2**). Active caspase-1 was encapsulated in the microvesicles as observed by WEHD assay (**Table 5** **, Panel 3**). Increased caspase-3 activity was also observed in VSMCs upon cell death induction (**Table 5** **, Panel 4**).

**Table 5:** Time course of VSMC cell death by MV.

*Time*	*Annexin-V assay*	*CV assay*	*Casp-1 activity in MV*	*Casp-3 activity in MV*
(h)	(%positive cells)	(% cell death	(AFU/mg)	(AFU/mg)
0	7	1±0.9	0	0.2
0.25	42	40±3	11.6	4.6
0.5	50	48±4	16.8	7.8
1	54	53±3	17.3	16.1
2	60	58±8	10.0	12.8
4	65	62±4	23.4	15.2
6	61	64±3	18.4	16.3
12	57	65±3	14.5	12.1
24	54	58±3	9.4	8.3

ˆMonocytes were stimulated with LPS for indicated period of time and microvesicles (MV) were isolated. MVs were then subjected to VSMC and analyzed for cell death using Annexin-V assay **(Panel 1)** and Crystal violet (CV) assay **(Panel 2)**. Caspase-1 activity **(Panel 3)** and caspase-3 activity **(Panel 4)** were measured from MVs and VSMCs respectively. Annexin-V and caspase -1 and 3 assays represented here are average of N = 2 experiments. CV assay is representative of N = 4 experiments.

**Figure 3:**
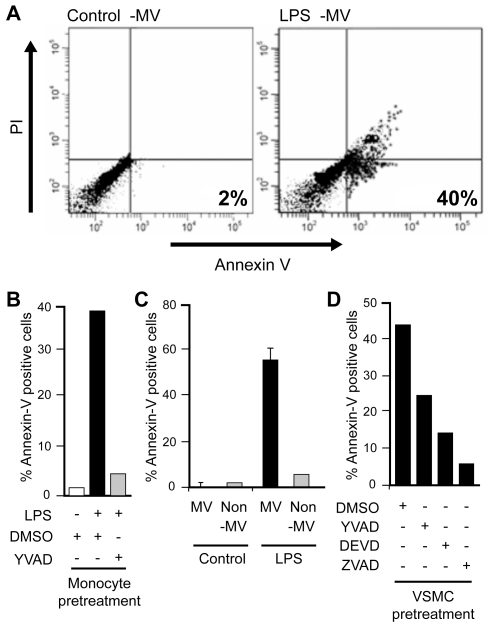
Microvesicles released by LPS- stimulated monocytes induce VSMC apoptosis. Monocytes were stimulated with LPS (1 µg/ml) for 15 min and microvesicles were isolated from supernatant by ultracentrifugation. VSMCs were then treated with microvesicles isolated from unstimulated, LPS, and LPS + YVAD treated monocytes. Cell death was measured by Annexin V/PI assays using flow cytometry. A) Representative data of apoptosis of VSMC by flow cytometry using Annexin V/PI assay. B) Average of quantitative analysis of the same (n = 2); C) VSMCs were pretreated with specific caspase inhibitors, YVAD (casp-1inhibitor), DEVD (casp-3 inhibitor) and ZVAD (pan-caspase inhibitor). MVs were isolated from monocytes stimulated with LPS for 15 min and subjected to the pretreated VSMCs for 18 h. Cell death was analyzed by Annexin V/PI assays (n = 2); D) Monocytes were again stimulated with LPS (1 µg/ml) for 15 min and both microvesicles (MV) and the non-vesicular fractions (non-MV) were isolated from supernatant of monocytes using ultracentrifugation. Both microvesicle (MV) and non-microvesicle (non-MV) fractions were subjected to VSMC and apoptosis was analyzed using Annexin V/PI assays.

To test the involvement of encapsulated caspase-1 in vesicles in VSMC cell death induction, MVs were isolated from monocytes either pretreated or not with caspase-1 inhibitor, YVADcmk prior to LPS challenge for 15 min. Increased cell death of VSMC (approximately 40%) was observed as compared to vesicles from unstimulated monocytes (**Figure 3A**). Vesicles from monocytes pretreated with YVADcmk before LPS challenge were unable to induce death of VSMC (**Figure 3B** **and** **Table 1** **, Second panel**), suggesting the possible role of caspase-1 or its substrate in the induction of VSMC cell death. The non-vesicular fractions did not induce killing of the smooth muscle cells (**Figure 3C**). To further investigate the possibility of caspase-1 and other caspase involvement in this cell death induction, VSMCs were pretreated with YVAD (caspase-1 inhibitor), DEVD (caspase-3 inhibitor) and ZVAD (pan-caspase inhibitor) before being subjected to MVs isolated from monocytes stimulated with LPS for 15 min. Cell death was measured after 18 h. All caspase inhibitors were capable of inhibiting induction of VSMC cell death. YVAD abrogated approximately 50% of cell death induced by MVs, whereas DEVD and ZVAD prevented 70% and 90% respectively (**Figure 3D** **and** **Table 1** **, Third panel**).

**Figure 4:**
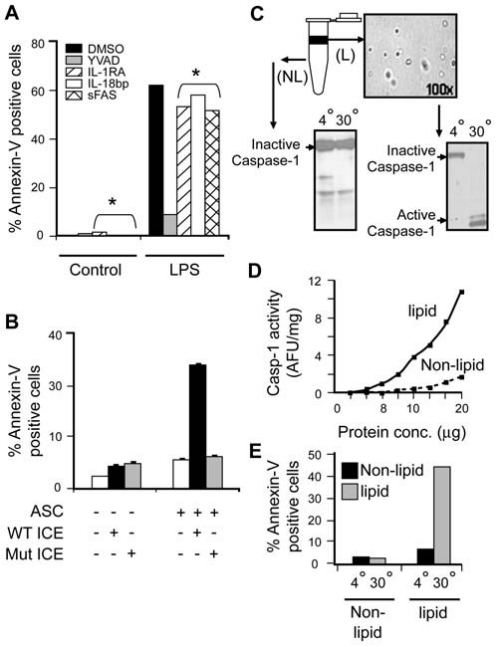
Microvesicle encapsulated exogenous caspase-1 directly induces VSMC apoptosis. A) Monocytes were either pretreated with YVAD-cmk or DMSO for 30 min. Cells were then rinsed with PBS and then stimulated with LPS (1 µg/ml) for 15 min. Microvesicles were isolated from the conditioned medium. Additionally, VSMCs were also pretreated with either saline, or with IL-1RA, IL-18 bp or sFAS for 30 min (* indicates VSMC pretreatments). VSMCs were then subjected to the isolated microvesicles from LPS stimulated monocytes. (▪) VSMC+ MV from control monocytes; (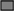) VSMC+ MV from LPS/YVAD monocytes; (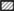) VSMC treated with IL-1RA+ MV from LPS treated monocytes; (□) VSMC treated with IL-18 bp + MV from LPS treated monocytes; (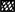) VSMC treated with sFAS+ MV from LPS treated monocytes. Apoptosis of VSMC was measured using Annexin V/PI assay. Graph represents average of n = 2 experiments. B) HEK-293 cells were transfected with either wild type or mutant caspase-1 (0.2 µg) along with ASC (0.2 µg) using Lipofectamine 2000. Transfection was normalized using vector controls. Supernatants were collected from the transfected cells and subjected to VSMC. Apoptosis was measured using Annexin V/PI assays. THP-1 cells were lysed and cell lysates were either kept at 4°C or incubated at 30°C for 1 h. Cell lysates were then centrifuged at 15,000 X g for 20 min. Both lipid and non-lipid fractions were measured for caspase-1 activity using C) immunoblot and D) WEHD-enzymatic assay. E) Both fractions were also subjected to VSMC and cell death was analyzed by Annexin V/PI assays.

To test if the LPS–induced monocyte-specific killing of smooth muscle cells was directly due to the exogenous caspase-1, as opposed to its products, we used blocking reagents to common cytokines released by monocytes upon LPS challenge (IL-18 and IL-1β) as well as Fas ligand to then monitor VSMC killing. Briefly, monocytes were pretreated with specific caspase-1 inhibitor (YVADcmk) or DMSO for 30 min. Monocytes were then washed with PBS to remove the remaining inhibitor. VSMC cells were also pretreated with IL-1 receptor antagonist (IL-1RA), IL-18 binding protein (IL-18BP), soluble Fas ligand or saline (as control) for 30 min. Monocytes were then stimulated with LPS for 15 min and microvesicles were isolated from the conditioned medium. Pretreated VSMCs were then subjected to these microvesicles (as described earlier) and analyzed for cell death. VSMC death was abrogated only by pretreatment with the specific caspase-1 inhibitor, YVADcmk. Inhibiting IL-18, IL-1β or FAS did not significantly affect the killing of VSMC, as compared to saline control (**Figure 4A**). We also performed experiments stimulating vascular smooth muscle cells with LPS, rIL-18, IL-1β or soluble FasL directly for 24 h. Direct stimulation of smooth muscle cells with these stimuli did not induce VSMC apoptosis.

As an alternate approach to confirm the specific role of exogenous active caspase-1 in inducing smooth muscle death, plasmids expressing EGFP tagged pro-caspase-1 (both cysteine wildtype and mutant) were expressed in HEK293 cells along with the inflammasome protein ASC. ASC is capable of activating the wild type caspase-1 but not the cysteine active site mutant of caspase-1. Expression patterns of wild type and mutant caspase-1 as well as ASC in HEK293 cells were confirmed by immunoblot. Activity of caspase-1 upon transfection was also confirmed by enzymatic assay and immunoblotting (data not shown). Smooth muscle cells were then cultured in conditioned medium from both active and mutant caspase-1 transfected cells. Conditioned medium containing wildtype active caspase-1 was capable of inducing apoptosis but the mutant form was not, confirming the specific role of active caspase-1 in inducing cell death (**Figure 4B**).

This novel role of caspase-1 was further confirmed by using a THP-1 derived cell free system. Cell free extracts from THP-1 cells were incubated either at 4°C or 30°C for 1 h and then subjected to centrifugation at 10,000xg following the caspase-1 activation protocol of Dubyak *et al* [30]. Active caspase-1 was identified only in the lipid layer of the cell free extract after incubation at 30°C (**Figure 4C**). Activity was also confirmed using WEHD-afc cleavage (**Figure 4D**). The lipid layer exhibited a significant amount of caspase-1 activity when compared to non-lipid fractions. When smooth muscle cells were incubated with either the lipid or non-lipid extract of THP-1 either kept at 4°C or incubated at 30°C, only lipid fractions having active caspase-1 induced smooth muscle cell apoptosis (**Figure 4E**).

### Encapsulation is necessary for exogenous caspase-1 induced cell death

**Figure 5:**
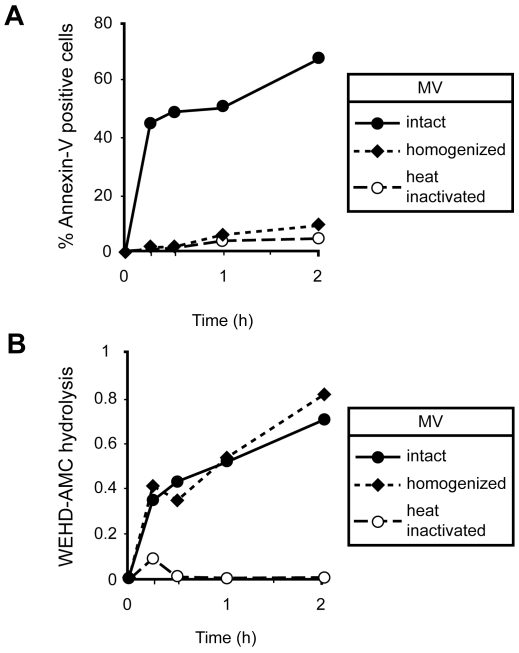
Vesiculation is necessary for exogenous caspase-1 mediated apoptosis of VSMC. Monocytes were either stimulated with LPS (1 µg/ml) for 15 min, 30 min, 1 h and 2 h or left untreated prior to microvesicles isolation. Microvesicles were then either kept intact or disrupted by mild homogenization or subjected to heat inactivation of encapsulated caspase-1. A) Microvesicles isolated from different times of LPS stimulation were then subjected to VSMC for 18 h and induction of apoptosis of VSMC was analyzed by Annexin V/PI assay. B) Caspase-1 activity of each fraction was measured using WEHD enzymatic assay. Time (h) in the figure indicates time of LPS stimulation of monocytes prior to MV isolation.

Finally, we examined the role of encapsulation in the cell death induced by exogenous caspase-1. Since caspase-1 release from stimulated monocytes was contained in microvesicles, we asked whether this encapsulation was critical for the cell death induction. To test this hypothesis, microvesicles derived from LPS stimulated monocytes (as described previously) were either kept intact or subjected to disruption (15 strokes on ice) or heat-inactivation (incubated at 65°C for 10 min). VSMCs were then cocultured with these vesicles and analyzed for cell death by Annexin V and crystal violet assays. Intact microvesicles from LPS stimulated monocytes induced significant VSMC cell death (∼50% as compared to cells exposed to vesicles shed by unstimulated monocytes by Annexin V assay) (**Figure 5A)**. The crystal violet assay demonstrated a similar trend of cell death; VSMCs treated with intact microvesicles exhibited approximately 50% cell death as compared to 2–10% by homogenized and heat inactivated microvesicle treatments (**Table 1** **, Bottom panel**). In contrast, cell death was completely abrogated in cells exposed to both ruptured and heat-inactivated microvesicles. Intact, homogenized and heat-inactivated microvesicles were then analyzed for caspase-1 activity using both immunoblot and enzymatic assay systems. Both intact and homogenized microvesicles contained active caspase-1, whereas the caspase-1 activity was completely lost by heat-inactivation of microvesicles (**Figure 5B**).

## Discussion

Executioner caspases such as caspases 3, 6 and 7 are recognized mediators of apoptotic cell death [4], [31]–[33], whereas caspase-1 is the prime member of the inflammatory caspase family which functions to activate proIL-1β and proIL-18. Nevertheless, caspase-1 clearly can contribute to cell death as has been described for macrophages responding to intracellular pathogens in pyroptosis [14]–[17] and as we have shown for splenic B lymphocyte apoptosis in response to sepsis [14]. However, that caspase-1 might be released from one cell to induce the cell death of a bystander cell has not been previously described. It is in this context that we here describe a novel pathway of caspase-1 mediated cell death. Monocytes induced by endotoxin are capable of releasing caspase-1 encapsulated in microvesicles that has the capacity to target an external cell, in this case proliferating vascular smooth muscle cells, for programmed cell death.

VSMC apoptosis occurs in all stages of atherosclerosis and is triggered by a combination of factors and conditions [18]–[21]. Because monocytes are the key inflammatory cells present in atherosclerotic plaques, we hypothesized that monocytes might provide the stimulus for VSMC apoptosis. We reasoned that this apoptotic event may be a critical component of atherosclerotic plaque rupture. We therefore developed an *in vitro* model of VSMC monocyte interactions to study potential mechanisms of the apoptotic event. Our findings document for the first time the novel role of monocyte-derived, microvesicular caspase-1 in the induction of VSMC apoptosis. Whereas monocytes cocultured with VSMC induced no significant cell death, remarkable VSMC death was induced by LPS treatment of the cocultures or when VSMCs were cocultured with conditioned medium from monocytes stimulated with LPS. Importantly, LPS in the absence of monocytes did not induce VSMC death. This apoptosis was completely abrogated when monocytes were pretreated with a specific caspase-1 inhibitor, YVADcmk.

Since it is generally believed that caspase-1's apoptotic function involves an enzymatic cleavage of cytosolic substrates, we wondered whether the cell death inducing activity from monocytes was packaged in vesicles. That is, encapsulation of caspase-1 would provide a means for both protecting the caspase-1 prior to cytosolic delivery and allowing its entry into the cytosol of targeted cells. Our findings support the concept that monocytes release microvesicles which carry active caspase-1 into the cytosol of targeted smooth muscle cells and induce apoptosis. We believe that our studies are the first to test this hypothesis.

Microvesicles have been known to be shed by cells during activation or apoptosis and to carry different factors and proteins. Monocyte/macrophage derived MVs have been reported to transport biologically significant amounts of phosphatidylserine and tissue factor [23]–[25], [34], [35]. Of note, monocyte/macrophage derived MVs have been found in human atherosclerotic lesions [23], [34], [36]. Furthermore, caspase-1 has also been reported to be enhanced in the plaque regions associated with an increase in infiltration of monocytes and macrophages [36].

Whether this encapsulation of active caspase-1 is important in the actual events related to plaque rupture remain to be shown. However, our data provide clear evidence to support the packaging of exogenous caspase-1 into microvesicles. These particles have the potential to contribute to smooth muscle cell death in plaques. Interestingly, the release of caspase-1 in vesicles parallels what we know about the release of the caspase-1 substrates IL-1β and IL-18. IL-1β release has been hypothesized to occur in the form of small packages termed microvesicles or vesicular bodies [26]–[29]. It is therefore, reasonable to hypothesize that caspase-1 release from cells might also require vesiculation. In this context, the present study confirms that active caspase-1 is released by activated monocytes in microparticles less than 0.1 µm and that this encapsulation is critical for its apoptotic function. Support for the need for caspase-1 encapsulation derives from our finding that mechanical disruption of microvesicles completely abrogates their apoptotic potential.

Members of the caspase-related protease family have been shown to play an important role in apoptosis [4], [14]–[17], [37]–[40]. However the specific role of caspase-1 in apoptosis is controversial. Caspase-1 knockout animals are born healthy without detectable morphological abnormalities, whereas caspase-3 deficient animals have major birth defects, particularly neurological defects which imply a role for caspase-3 in developmental apoptosis [39], [40]. Furthermore, we have previously documented that spontaneous monocyte apoptosis is not dependent upon caspase-1 but upon caspase-3 activity [41]. On the other hand, over expression of caspase-1, in a rat fibroblast cell line, induces an apoptosis which is blocked by crmA, a cow pox virus protein that inhibits caspase-1 [42]. The involvement of caspase-1 in neuronal cell apoptosis is also well established. Gagliardini *et al*, 1994, observed the ability of a caspase-1 inhibitor to prevent apoptosis induced by nerve growth factor deprivation [43]. Furthermore, caspase-1 has been implicated in the death of Salmonella infected dendritic cells and monocyte derived macrophages [43]. Work from our own laboratory has shown the unique role of caspase-1 in regulating sepsis survival by regulating lymphocyte apoptosis [14], [44]. Thus, the present work lends support to the notion that caspase-1 is important in at least selected forms of programmed cell death. Our findings suggest that caspase-1 directly regulates the apoptosis of smooth muscle cells and that encapsulation of this active caspae-1 in microvesicles is critical for its function.

Other potential mechanisms to explain the VSCM apoptosis by the monocyte product include the concomitant release of potent cytokines. Caspase-1 is known to be principally responsible for the production of mature IL-1β and IL-18 from their precursor forms. These inflammatory cytokines have been linked to organ injury and failure [14], [44], [45]. However, in this context, we found no statistical difference between apoptosis of smooth muscle cells that were subjected to conditioned medium after blockade of both IL-1β and IL-18.

Lastly, and perhaps linked to the apoptosis concept, is the possibility that caspase-1 has important effects on the host intracellular defense system. This idea is supported by the growing body of evidence that places caspase-1 at the center of complex of regulatory proteins termed the inflammasome [6], [46]. It is believed that caspase-1 interacts with various members of these novel molecules via its caspase recruitment domain or CARD [6]–[8]. It is likely that caspase-1 may play a central role in the structural integrity, organization and/or regulation of these intracellular protein complexes. For example, caspase-1 is known to interact with RIP2, a kinase that is important in upstream activation of NF-κB, via CARDs on both molecules [47], [48]. We have recently demonstrated that caspase-1 may also function as a scaffolding molecule that promotes RIP2 mediated NF-κB activation [47].

In summary, our results demonstrate that caspase-1 plays a central role in the regulation of apoptosis. This novel apoptotic event is dependent upon encapsulation of exogenous caspase-1 into released microvesicles that allow targeting of the active enzyme to the cytosol of different cells, in this study vascular smooth muscle cells. Future studies will need to determine whether this mechanism is targeted to specific cells or acts via a concentration dependent stochastic process.

## Materials and Methods

### Reagents

Bacterial lipopolysaccharide (LPS), Escherichia coli strain 0127:B8, Westphal preparation, was obtained from Difco (Detroit, MI). RPMI 1640 and phosphate buffered saline (PBS) was purchased from BioWhitaker (Walkersville, MD), and fetal bovine serum (FBS) was obtained from Hyclone (Logan, UT). The pan-caspase inhibitor, z-Val-Ala-Asp (O-Methyl) fluoromethyl ketone (zVADfmk) and inactive caspase inhibitor, z-Phe-Ala-fluoromethylketone (zFAfmk) were purchased from Enzyme Systems, (Irvine, CA). All other reagents were obtained from Sigma-Aldrich (St. Louis, MO) unless otherwise specified.

### PBMC isolation and culture conditions

Human peripheral blood monocytes were isolated from the heparinized blood of normal donor or buffy coats from the American Red Cross. Normal donor blood samples were obtained after informed written consent as approved by the Ohio State Institutional Review Board. Details provided in online supplement. Isolated monocytes were cultured in a 5-ml polypropylene tube at 10×10^6^ cells/ml in RPMI1640 supplemented with 10% fetal bovine serum (FBS) at 37°C in humidified incubator. Typically, cells were stimulated with LPS (1 µg/ml) for indicated time and the cell pellet separated from supernatant by centrifugation at 3000 X g for 5 min.

### Vascular smooth muscle cell culture

Vascular smooth muscle cells (VSMCs) (passage 3 to 7) were obtained from Clonetics (Biowhitaker, Inc, Walkersville, MD) from human heart donors. VSMC identity was initially confirmed by α-smooth muscle actin staining. VSMC initially were grown in growth medium (SmGM2; Clonetics) with 5% FBS, and at 60–70% cell confluence, the media was changed to serum-free medium (50∶50 of DMEM/F12 media with 5 ml of ITS, PSA, L-glutamine, and nonessential amino acids per 500 ml of solution). VSMC were co-cultured with either monocytes or conditioned medium from monocytes as per further experiments. Co-culture experiments were performed with RPMI1640 in 10% FBS throughout the experiments.

### Cell death assays

VSMC cells were seeded in cell culture plates at a density of 60–70% confluency of cells/well. After overnight incubation with monocytes, conditioned medium or microvesicles from conditioned medium in the presence or absence of inhibitors, VSMC were subjected to cell death analysis. Cell death was identified using light microscopy, Annexin V, caspase-3 activation [49] and crystal violet and LDH assays, as described in details in supplement information (Appendix S1).

### Microvesicle isolation and identification

Microvesicles were isolated from conditioned medium using ultracentrifugation as described in SI. Isolated microvesicles were characterized using flow cytometry and transmission electron microscopy.

### ELISAs

Cytokine levels of IL-1β and IL-18, as well as caspase-1 levels were measured by sandwich enzyme linked immunoassay (ELISA) (details in Appendix S1).

### Caspase-1 activity

For caspase-1 activity assay, monocytes were isolated from buffy coats and cultured at a concentration of 10×10^6^ cells/150 µl in 96-well plates. Cells were then stimulated with LPS (5 µg/ml) and supernatants were collected from each well. Cells were spun at 1000g×4°C for 10 min to collect the supernatant. Supernatants were then subjected to caspase-1 ELISA or enzymatic assay [49]–[51]. Assay details in supplement section (Appendix S1).

### In-vitro caspase-1 activation

In-vitro caspase-1 activation was performed using THP-1 cells as described by in Appendix S1.

### Expression plasmids, cell culture and transfection

Caspase-1, ASC plasmids were either created or obtained as gifts. Inhibitors of caspase-1 were purchased. HEK293 cells were cultured and used for transfection of these plasmids. See details in Appendix S1.

### Statistical analysis

Data are represented as the mean ± standard error of the mean (SEM) from at least three independent experiments. All other simple comparisons were performed with Student's *t* test, with p<0.05 considered to represent statistical significance.

## Supporting Information

Appendix S1 (0.04 MB DOC)
