# Supplementary material for: Monocyte Derived Microvesicles Deliver a Cell Death Message via Encapsulated Caspase-1
Source: PLoS One. 2009 Sep 25;4(9):e7140. doi: 10.1371/journal.pone.0007140 (PMC2744928; doi:10.1371/journal.pone.0007140)
Supplement: Appendix S1 — (0.04 MB DOC) [file pone.0007140.s001.doc]

# Materials and Methods:

## *Reagents*

Bacterial lipopolysaccharide (LPS), Escherichia coli strain 0127:B8, Westphal preparation, was obtained from Difco (Detroit, MI). RPMI 1640 and phosphate buffered saline (PBS) was purchased from BioWhitaker (Walkersville, MD), and fetal bovine serum (FBS) was obtained from Hyclone (Logan, UT). The pan-caspase inhibitor, z-Val-Ala-Asp (O-Methyl) fluoromethyl ketone (zVADfmk) and inactive caspase inhibitor, z-Phe-Ala-fluoromethylketone (zFAfmk) were purchased from Enzyme Systems, (Irvine, CA). All other reagents were obtained from Sigma-Aldrich (St. Louis, MO) unless otherwise specified.

## *PBMC isolation and culture conditions*

Human peripheral blood monocytes were isolated from the heparinized blood of normal donor or buffy coats from the American Red Cross. First, peripheral blood mononuclear cells (PBMC) were isolated by using HIstopaque-1077 density gradient. Monocytes were further purified with a system of MACS CD14 Microbeads (Miltenyi Biotec, Auburn, CA) selecting CD14+ cells. Isolated monocytes were cultured in a 5-ml polypropylene tube at 10 X 10^6^ cells/ml in RPMI1640 supplemented with 10% fetal bovine serum (FBS) at 37^o^C in humidified incubator. Typically, cells were stimulated with LPS (1μg/ml) for indicated time and the cell pellet separated from supernatant by centrifugation at 3000 X g for 5 min.

## *Vascular smooth muscle cell culture*

Vascular smooth muscle cells (VSMCs) (passage 3 to 7) were obtained from Clonetics (Biowhitaker, Inc, Walkersville, Md) from human heart donors. VSMC identity was initially confirmed by α-smooth muscle actin staining. VSMC initially were grown in growth medium (SmGM2; Clonetics) with 5% FBS, and at 60-70% cell confluence, the media was changed to serum-free medium (50:50 of DMEM/F12 media with 5ml of ITS, PSA, L-glutamine, and nonessential amino acids per 500ml of solution). VSMC were co-cultured with either monocytes or conditioned medium from monocytes as per further experiments. Co-culture experiments were performed with RPMI1640 in 10% FBS throughout the experiments.

## *Cell death assays*

VSMC cells were seeded in cell culture plates at a density of 60-70% confluency of cells/well. After overnight incubation with monocytes, conditioned medium or microvesicles from conditioned medium in the presence or absence of inhibitors, VSMC were subjected to cell death analysis. Dying cells were identified first by light microscopy. Cells were judged to be apoptotic when they exhibited the characteristic features of shrunken, compacted nuclei (pyknosis) and/or nuclear fragmentation (karyorrhexis). A minimum of ten random fields was examined at 100x magnification and the numbers of apoptotic features were recorded. Smooth muscle cell death/apoptosis were also analyzed by Annexin V and caspase-3 assays (as a marker for cellular death). The annexin V assay was performed using apoptosis detection kits purchased from BD Biosciences Pharmingen, San Diego, CA following the manufacturer’s protocol. Active caspase-3 was quantified by cleavage of the peptide substrate asp-glu-val-asp-aminotrifluoromethylcoumarin (DEVD-afc), as previously described (34, 35). Briefly, cells were snap frozen in liquid nitrogen and lysed by four cycles of freeze thawing. Lysates were then incubated with DEVD-afc in a cyto-buffer (10% glycerol, 50mM PIPES, pH 7.0, 1mM EDTA) containing 1mM DTT and 20μM tetrapeptide substrate (Enzyme Systems Products, Livermore, CA). Release of free afc was determined using a Cytofluor 4000 fluorimeter (Filters: excitation; 400 nm emission; 508 nm; Perseptive Co., Framingham, MA). Cell death was also analyzed using crystal violet assay. Briefly, culture medium was removed from cells and washed with PBS warmed at room temperature. Cells were then incubated with 0.2% crystal violet solution for 10 minutes at room temperature. Cells were then gently washed with water (avoiding washing off cells). 1% SDS solution was then added to solubilize the stained cells by gentle agitation on orbital shaker. Absorbance is read at 570nm. Finally cytotoxicity due to cell death was also measured using LDH assay. LDH was measured using cytotoxicity detection kit from Roche Applied Science, Germany following manufacturer’s protocol.

## *Microvesicle isolation and identification*

Conditioned medium was first centrifuged at 1000 X g for 5 min and then at 15,000 X g for 15 min to remove cells, cell debris and aggregates. The supernatant was then ultracentrifuged at 100,000 X g for 1h. Pelleted vesicles then washed by resuspending them in PBS and again spinning at 100,000 g for 1h (27). Microvesicles were then subjected to characterization using flow cytometry and transmission electron microscopy (TEM). Briefly, microvesicles were sized by comparison to calibrations of the flow cytometer using beads ranging from <0.1-1 micron from Spherotech Inc, IL using the manufacture’s protocol.

Microvesicles were also characterized using transmission electron microscopy. Briefly, isolated microvesicles were resuspended in saline and fixed using 1% phosphotungstic acid. Microvesicles were incubated for 2 min and placed on grids for observation under FEI Technai G2 Spirit transmission Electron Microscope (TEM). Pelleted microvesicles were then subjected directly to western blotting, caspase-1 enzymatic assay or added to VSMC.

## *ELISAs*

Cytokine levels of IL-1β and IL-18 were measured by sandwich enzyme linked immunoassay (ELISA). Briefly, the mature IL-1β ELISA used the mAb clone 8516 (R & D Systems, Minneapolis, MN) and rabbit polyclonal mature IL-1β Ab (raised against entire 17-kDa mature IL-1β) as capture and detection antibodies, respectively. Streptavidin-conjugated HRP (Amersham Pharmacia Biotech, Piscataway, NJ) and TMB Microwell Peroxidase Substrate System (Kirkegaard & Perry, Gaithersburg, MD) were used for quantitaion. For the measurement of IL-18, ELISA kits from R&D Systems were used. Total caspase-1 released in supernatants was also measured using caspase-1 ELISA. Briefly, goat polyclonal caspase-1 Ab (G273, Covance, CA) and rabbit polyclonal caspase-1 Ab (raised against mature caspase-1) were used for capture and detection antibodies, respectively. Goat anti-rabbit streptavidin-conjugated HRP (Amersham Pharmacia Biotech, Piscataway, NJ) was used for quantification. Caspase-1 quantification was arbitrarily defined by the amount of caspase-1 present in monocytes as we have previously observed, i.e., 3 ng/10^6^ fresh human peripheral blood monocytes (51).

## *Caspase-1 activity*

For caspase-1 activity assay, monocytes were isolated from buffy coats and cultured at a concentration of 10 X 10^6^ cells /150μl in 96-well plates. Cells were then stimulated with LPS (5μg/ml) and supernatants were collected from each well. Cells were spun at 1000g X 4^o^C for 10 min to collect the supernatant. Supernatants were then subjected to caspase-1 ELISA or enzymatic assay. 100μl of supernatant were mixed with 50μl of an assay buffer (50mM HEPES (pH 7.4), 100 mM NaCl, 0.1% 3-[(3-cholamidopropyl)dimethylammonio]-1-propanesulfonate, 20% glycerol, 10mM DTT and 0.1mM EDTA) and 5μl of 1mM Ac-WEHD-AMC (50). This mixture was placed in a well of a Costar 96-well flat bottom plate (Corning Glass, Corning, NY) and immediately subjected to kinetic fluorometric assay using a Cytofluor 4000 fluorometer (Perspective, Framingham, MA) with filters of 360nm excitation and 460nm emission for 2h at room temperature. The linear change of the fluorescence of hydrolyzed free AMC per time and the protein concentrations of the assayed samples were used for calculating caspase activity with a conversion factor obtained from the assay for constructing an AMC standard curve.

## *In-vitro caspase-1 activation*

1 X 10^8^ THP-1 were resuspended in 1ml of buffer W (20mM HEPES, pH 7.5, 1.5mM MgCl2, 1mM EGTA, 1mM EDTA) supplemented with 2mM DTT, 2μg/ml leupeptin, 100 μg/ml PMSF and 2.5 μg/ml aprotinin. The cells are then pelleted, all but approximately 50μl of the buffer was removed. The cells were then allowed to swell for 10min on ice and were subsequently lysed by 15 passages through a 22G needle (30). Lysates were spun at 15,000 X g for 15min. Both lipid and non-lipid fractions of the supernatant were separated and either kept on ice or further incubated at 30^O^C. Fractions were then subject to either immunoblot, enzymatic or functional assays.

***Expression plasmids, cell culture and transfection***

Caspase-1 plasmid was a gift of Merck Research Laboratory. ASC was cloned from human monocyte cDNA and confirmed by DNA sequencing. A catalytically inactive caspase-1 (mutated at cysteine 285) was created and confirmed by DNA sequencing. Green fluorescent protein (GFP) fusion vectors were generated by subcloning into pEGFP expression vectors (Clontech Laboratories, Palo Alto, CA). Caspase-1 inhibitor Ac-Tyr-Val-Ala-Asp-chloromethyl ketone (YVAD-cmk) was purchased from Calbiochem, San Diego, CA). HEK293 cells were cultured in DMEM supplemented with 10% heat-inactivated FBS. HEK293 cells were transfected using Lipofectamine 2000 (Invitrogen life technologies, Carlsbad, CA) according to the manufacturer’s recommendation. Expression of caspase-1 and ASC were confirmed using immunoblot.

### *Statistical analysis*

Data are represented as the mean + standard error of the mean (SEM) from at least three independent experiments. All other simple comparisons were performed with Student’s *t* test, with p<0.05 considered to represent statistical significance.
